# The MULTICOM toolbox for protein structure prediction

**DOI:** 10.1186/1471-2105-13-65

**Published:** 2012-04-30

**Authors:** Jianlin Cheng, Jilong Li, Zheng Wang, Jesse Eickholt, Xin Deng

**Affiliations:** 1 Department of Computer Science, University of Missouri-Columbia, Columbia, MO, 65211, USA; 2 Informatics Institute, University of Missouri-Columbia, Columbia, MO, 65211, USA; 3 C. Bond Life Science Center, University of Missouri-Columbia, Columbia, MO, 65211, USA

**Keywords:** Protein structure prediction, Bioinformatics tool, Secondary structure, Solvent accessibility, Domain, Contact map, Tertiary structure, Protein model quality assessment, Fold recognition, Protein disorder

## Abstract

**Background:**

As genome sequencing is becoming routine in biomedical research, the total number of protein sequences is increasing exponentially, recently reaching over 108 million. However, only a tiny portion of these proteins (i.e. ~75,000 or < 0.07%) have solved tertiary structures determined by experimental techniques. The gap between protein sequence and structure continues to enlarge rapidly as the throughput of genome sequencing techniques is much higher than that of protein structure determination techniques. Computational software tools for predicting protein structure and structural features from protein sequences are crucial to make use of this vast repository of protein resources.

**Results:**

To meet the need, we have developed a comprehensive MULTICOM toolbox consisting of a set of protein structure and structural feature prediction tools. These tools include secondary structure prediction, solvent accessibility prediction, disorder region prediction, domain boundary prediction, contact map prediction, disulfide bond prediction, beta-sheet topology prediction, fold recognition, multiple template combination and alignment, template-based tertiary structure modeling, protein model quality assessment, and mutation stability prediction.

**Conclusions:**

These tools have been rigorously tested by many users in the last several years and/or during the last three rounds of the Critical Assessment of Techniques for Protein Structure Prediction (CASP7-9) from 2006 to 2010, achieving state-of-the-art or near performance. In order to facilitate bioinformatics research and technological development in the field, we have made the MULTICOM toolbox freely available as web services and/or software packages for academic use and scientific research. It is available at http://sysbio.rnet.missouri.edu/multicom_toolbox/.

## Background

The central dogma of protein science is that protein sequence specifies protein structure; and protein structure determines protein function. Therefore, understanding protein structure is crucial for elucidating protein function and has fundamental significance in biomedical sciences including protein function analysis, protein design, protein engineering, genome annotation, and drug design. Since the experimental determination of the first two protein structures - myoglobin and haemoglobin - using X-ray crystallography [1,2], the structures of more and more proteins have been solved by either X-ray crystallography or Nuclear Magnetic Resonance (NMR) techniques. Currently, there are about 75,000 protein sequences with determined structures deposited in the Protein Data Bank (PDB), which account for about 0.07% of the total known protein sequences (i.e. > 108 million). With the exponential growth of protein sequences with unsolved structures produced by various high-throughput, next generation sequencing techniques, predicting protein structure from sequence, which is critical for filling the sequence-structure gap [3], has become one of the most fundamental problems in structural bioinformatics and genomics. Accurate high-throughput protein structure prediction tools are urgently needed for both scientific research as well as the bio-tech industry. These tools will also fulfill a very important and major goal of the structural genomics project, namely to provide a rather complete set of experimentally determined structures for predicting the structure of about 99.9% of proteins with unsolved structures [3].

The protein structure prediction problem is usually decomposed and attacked from the three different dimensional levels: 1D structure prediction, 2D structure prediction, and 3D structure prediction [4]. One-dimensional (1D) structure prediction is the prediction of protein structural features such as secondary structures, solvent accessibilities, disordered residues or domain boundaries along one-dimensional sequences. Since 1D prediction is usually the first step to obtain protein structure, the largest number of methods and tools had been developed for it, such as Porter [5], SAM [6], SSpro [7,8], PSIPRED [9], SABLE [10-13], YASSPP [14], Jpred [15], PREDATOR [16-18], and GOR [19] for secondary structure prediction; NetSurfP [20], ACCpro [7,21] and Real-SPINE [22] for solvent accessibility prediction; PONDR [23,24], MFDp [25], DISOPRED [26], SPINE-D [27], PrDOS [28], Spritz [8], POODLE [29-31], IUPRred [32,33], DISOclust [34], and IntFOLD-DR [35] for disorder prediction; DomPred [36], DomSVR [37], PPRODO [38], CHOPnet [39], DoBo [40] and SSEP-Domain [41] for domain boundary prediction; and PredictProtein [42], Distill [43], and SCRATCH [7] for all four kinds of 1D predictions.

Two-dimensional (2D) structure prediction is to predict the spatial relationships (e.g., residue-residue contacts, disulfide bonds, or beta-residue pairings) of two residues. 2D prediction is a challenging and increasingly important problem [44]. Some methods and tools for 2D prediction are PROFcon [45], Distill [43], TMHcon [46], DiANNA [47], GDAP [48], CYSPRED [49], BETAWRAP [50], SVM-BetaPred [44], BETTY [51], ProC_S3 [52], FragHMMent [53], SVMSEQ [54], and SAM [55].

Three-dimensional (3D) structure prediction is to predict the 3D coordinates of each residue [56-61], which is the ultimate goal of structure prediction. Some popular tools are I-TASSER [62-64], MODELLER [65,66], HHpred [67], QUARK [68], chunk-TASSER [69], Rosetta [61], Pcons-net [70], SAM [71], Raptor-X [72], SparksX [73], and MULTICOM. 1D, 2D, and 3D protein structure prediction methods are routinely evaluated in the Critical Assessment of Techniques for Protein Structure Prediction (CASP) [74] - a community-wide experiment for blind protein structure prediction that has been held every two years since 1994. CASP experiments have driven the development of protein structure prediction methods by objectively assessing the state of the art of the most active and imperative protein structure prediction problems. The last two CASPs (CASP8, 2008 and CASP9, 2010) [75] focused on trying to solve the most pressing structure prediction problems: disorder region prediction (1D) [76], residue-residue contact prediction (2D) [77], protein tertiary structure prediction (3D) [78-80], evaluation of 3D models [81-87], and protein model refinement [74,88,89].

During the last several years, we have developed a series of tools for predicting protein structure and structural features at the 1D, 2D, and 3D levels, including secondary structure prediction, solvent accessibility prediction, disorder region prediction, domain boundary prediction, contact map prediction, disulfide bond prediction, beta-sheet topology prediction, protein fold recognition, multiple template combination and alignment, protein tertiary structure modeling, protein model quality assessment, and mutation stability prediction. Most of these tools have been rigorously tested by many users in the last several years and/or during the last three rounds of the Critical Assessment of Techniques for Protein Structure Prediction (CASP7-9) achieving state-of-the-art or near performance. In order to facilitate bioinformatics research and technological development in the field, we have incorporated updates and improvements accumulated over years into these tools and packed them together into one single comprehensive MULTICOM toolbox equipped with tutorials, documentation, software executables, some source code, web service, and online mailing list for technical support.

**Figure 1:**
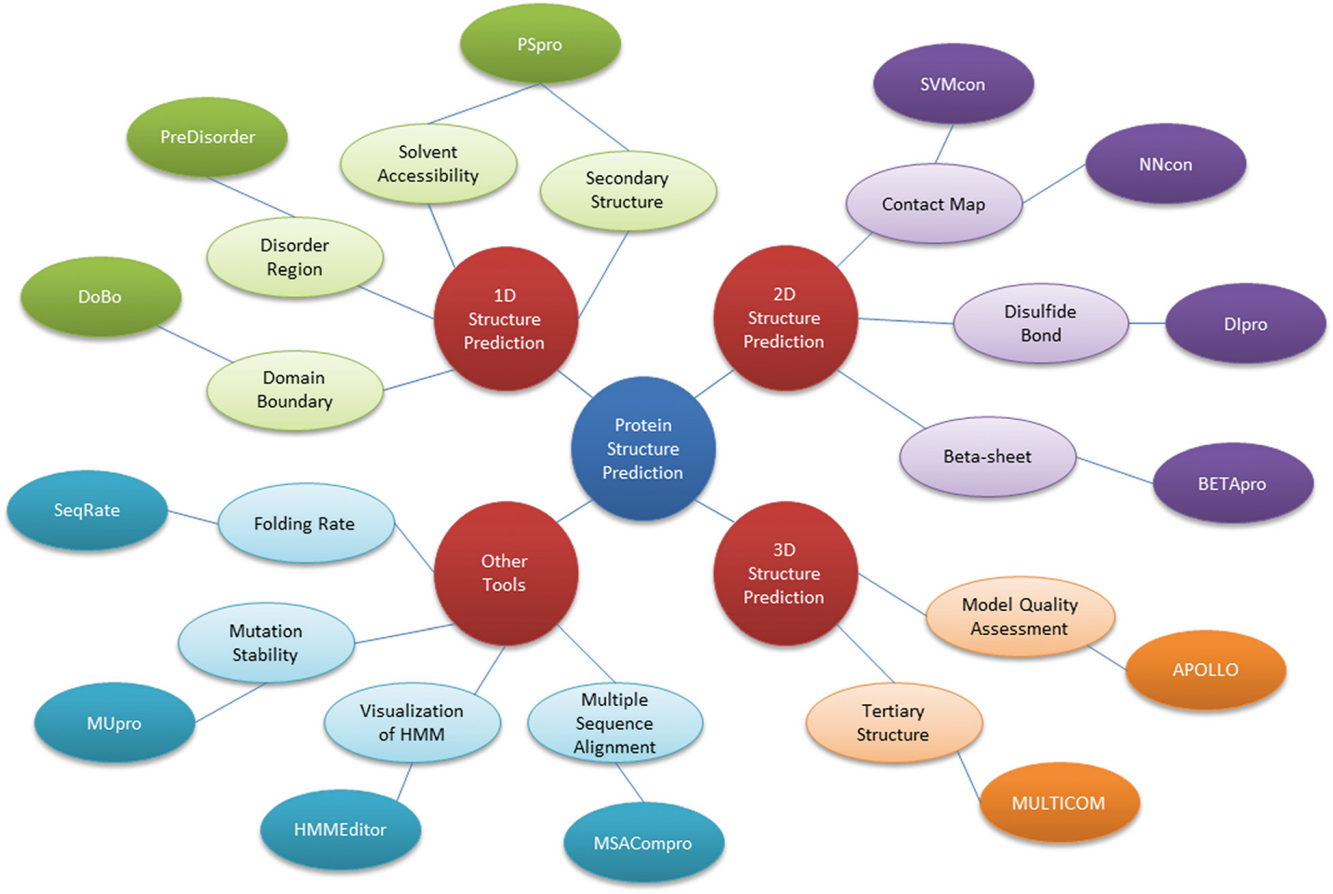
The organization of the MULTICOM toolbox.

The organization of the MULTICOM toolbox is shown in Figure 1. The 1D protein structure prediction tools are comprised of PSpro for the prediction of secondary structure and relative solvent accessibility, PreDisorder for disordered residue prediction, and DoBo for domain boundary prediction. The 2D protein structure prediction tools include SVMcon and NNcon for residue-residue contact prediction, DIpro for disulfide bond prediction, and BETApro for beta-sheet pairing prediction. The 3D protein structure prediction tools are comprised of MULTICOM for tertiary structure prediction and APOLLO for protein model quality assessment. The MULTICOM toolbox also contains several other protein bioinformatics tools including SeqRate for protein folding rate prediction, MUpro for the prediction of stability changes caused by single-residue mutation, MSACompro for multiple protein sequence alignment, and HMMEditor for visualization of protein Hidden Markov models. The entire MULTICOM toolbox is freely available for academic use and scientific research at http://sysbio.rnet.missouri.edu/multicom_toolbox/. Users may download and install most of the tools locally or access them through web services.

## Methods and benchmarks

### 1D structure prediction tools

#### *PSpro2.0 for secondary structure and relative solvent accessibility prediction*

PSpro2.0 is an improved and combined version of the popular tools SSpro/ACCpro 4 [7,8,21] for the prediction of protein secondary structure and relative solvent accessibility. It integrates both homology-based and *ab initio* methods to make predictions. The *ab initio* approach uses a 1-D recursive neural networks (1D-RNN) [7,90] and takes the profile of a query protein sequence as input to predict its secondary structures (i.e. helix, strand, and loop) or relative solvent accessibility (i.e. exposed and buried) at 20 different exposure thresholds (i.e. 0%, 5%, 10%, …, 95%). The sequence profile was generated by using PSI-BLAST to search the query sequence against a Non-Redundant protein (NR) sequence database, which has been updated to the most recent version. The PSpro2.0 allows users to plug in any version of the NR database of their choice.

The homology-based method in PSpro2.0 is called to make predictions if a significant homologous template protein can be found for a query protein in the Protein Data Bank (PDB) [91]. The homology-based method uses BLAST to search the query sequence against a locally compiled version of the PDB database to identify homologous hits. Information regarding the alignment between the query and the most significant hit, including the alignment e-value, the number of amino acids aligned, number of gaps, sequence identity, is gathered and used by a linear regression function to predict the accuracy of transferring the secondary structure and solvent accessibility of the hit to the query protein. The linear regression function was trained on a set of query-template alignments with known alignment information and transferring accuracy. If the predicted transferring accuracy is > = 0.82 for secondary structure (resp. > = 0.80 for relative solvent accessibility), the secondary structure (resp. relative solvent accessibility) is transferred from the hit to the query as predictions. Otherwise, *ab initio* predictions will be used. The combination of the *ab initio* method and homology-based method can automatically apply the most appropriate method for the query proteins having or not having significant homology with a known protein structure in order to improve the prediction performance. In order to take advantage of abundant new protein structures in the PDB, PSpro2.0 uses an updated local version of the PDB database comprised of 62,607 proteins. The new local PDB database is a few times larger than the old one used with SSpro/ACCpro 4 which had 22,064 proteins.

We benchmarked PSpro2.0 on the protein targets of the last two Critical Assessments of Techniques for Protein Structure Prediction (CASP8 in 2008 and CASP9 in 2010). The CASP datasets were chosen because of their wide adoption in the field, their balance of easy (homology-based) and hard (*ab initio or weak homology*) targets, and their relatively large size. When the homology-based method was tested, the target proteins in the CASP8 and CASP9 data sets were removed from the local PDB database in order to avoid using themselves to make predictions. 100 CASP9 targets and 119 CASP8 targets that were not present in the local PDB database were used in this test.

Table 1 reports the accuracy of secondary structure prediction and relative solvent accessibility prediction at a 25% threshold for both the combined method and the *ab initio* method alone. Here the accuracy is defined simply as the percent of correct predictions, i.e. the standard Q3 score for three-category secondary structure prediction, and the Q2 score for two-category relative solvent accessibility prediction. The results show that the accuracy of secondary structure prediction and relative solvent accessibility prediction of the combined method is in the range [80.8%, 83.3%] and [74.6%, 77.5%], respectively, higher than [76.6%, 77.7%] and [74.2%, 75.9%] of the *ab initio* method. Using homology prediction seems to improve secondary structure prediction more than relative solvent accessibility prediction. Combining homology and *ab initio* approaches seems to improve secondary structure prediction more than solvent accessibility prediction.

**Table 1:** The accuracy of the prediction of secondary structure (SS) and relative solvent accessibility (SA) on 100 CASP9 targets and 119 CASP8 targets, respectively

	**both *ab initio* and homology**		***ab initio* alone**	
**Dataset**	**SS**	**SA**	**SS**	**SA**
CASP8	83.30%	77.50%	77.73%	75.94%
CASP9	80.78%	74.56%	76.60%	74.20%

#### *PreDisorder1.1 for protein disorder prediction*

**Figure 2:**
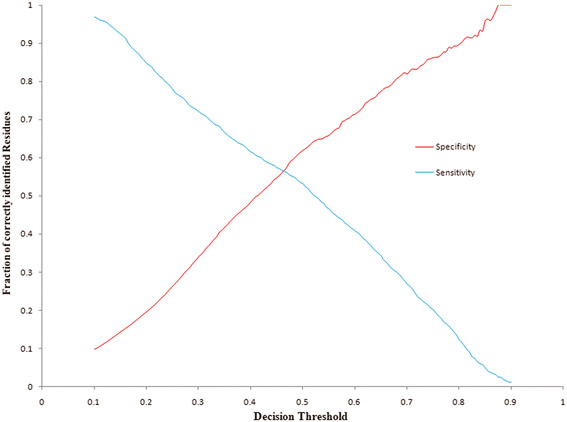
The plot of sensitivity and specificity (y axis) against different probability thresholds of classifying residues as disordered residues on CASP8 targets.

**Figure 3:**
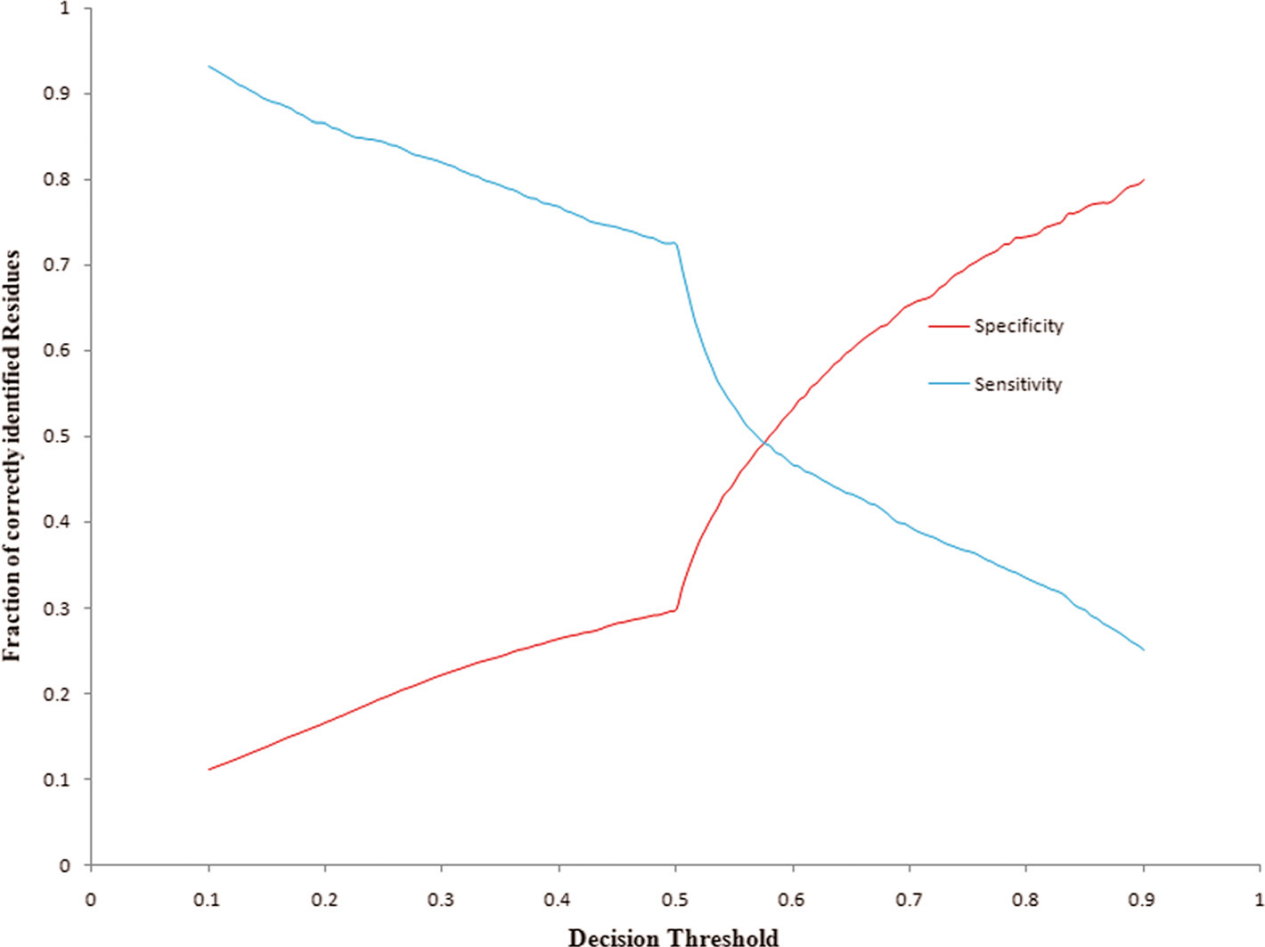
The plot of sensitivity and specificity (y axis) against different probability thresholds of classifying residues as disordered residues on CASP9 targets.

PreDisorder1.1 is an efficient and reliable *ab initio* prediction tool for protein disorder regions on the genomic scale. PreDisorder uses only sequence-related information in conjunction with neural networks to predict the disorder probability of each residue of a protein sequence. The earlier and most recent versions of PreDisorder had been consistently ranked as one of the top protein disorder predictors in the last three Critical Assessments of Techniques for Protein Structure Prediction (CASP7, 8, 9) in 2006, 2008, and 2010, respectively [92,93]. Evaluated on 117 CASP8 targets and 117 CASP9 targets separately, PreDisorder yielded an AUC score of 0.86 and 0.82, respectively [92,93]. AUC score represents the area under the Receiver Operating Characteristic (ROC) curve (true positive rates versus false positive rates) of disorder predictions. Considering different methods may use different criteria to set a probability threshold to make order/disorder decisions, we also calculated the break-even score and its corresponding decision threshold on predicted disorder probabilities. The break-even score is the value at which the sensitivity (i.e. recall) and specificity (i.e. precision) of disorder predictions are equal. The break-even scores on the CASP8 and CASP9 dataset are in the range [0.45, 0.56] using a probability threshold of around 0.5. Figures 2 and 3 illustrate the plots of sensitivity versus specificity over a varying decision threshold from 0.1 to 0.9 at step of 0.005 on the CASP8 and CASP9 data sets, respectively. The intersections in the figures denote the break-even points/scores.

#### *DoBo for protein domain boundary prediction*

Protein domain boundary prediction is often used as a means to decompose the modeling of a large, multi-domain protein in to smaller, more manageable pieces. In order for such a technique to be applicable to hard, free modeling targets it should not rely extensively on templates or known structures to delineate protein domain boundaries. DoBo [40] is the sequence based protein domain boundary predictor we have developed and included in the MULTICOM toolbox. It leverages evolutionary information contained in multiple sequence alignments to identify potential domain boundary sites. These candidate sites are then classified using a support vector machine. Predicted domain boundary sites are finally scored and a confidence value provided.

We recently evaluated DoBo on 14 continuous, multi-domain CASP9 targets [40]. DoBo is able to recall 70% of the domain boundaries, which occur at least 40 residues from the N or C terminal end of the sequence. The precision of the domain boundary prediction is 49%. Here, a domain boundary prediction is considered correct if it occurs within 20 residues of a true domain boundary. Furthermore, on a large benchmark dataset using a 10 fold cross validation procedure, DoBo achieves a break-even point of 60% (ie, precision equals recall) for domain boundary predictions [40].

### 2D structure prediction tools

#### *NNcon and SVMcon for general residue-residue contact prediction*

Residue-residue contact prediction continues to be an area of active research and becoming of greater importance in the latest rounds of CASP. Of particular importance to tertiary structure prediction are sequence based (ie *ab-initio*) contact prediction methods and recent work by Wu et al. has shown that predicted contact information can be used to significantly improve predictions for free modeling targets [94]. The MULTICOM toolbox contains two general residue-residue contact predictors – NNCon [95] and SVMcon [96]. NNcon [95] is a sequence-based, *ab initio* method to predict intra-chain protein residue-residue contacts. NNcon uses a set of two-dimensional (2D) recursive neural network ensembles [90] which predict the probability that the distance between any two residues are below a threshold (i.e. in contact). Features used for each residue include a sequence profile, secondary structure and solvent accessibility.

SVMcon [96] is an *ab initio* method based on a support vector machine (SVM). For each residue pair, a set of features including secondary structure, solvent accessibility and a sequence profile is encoded for a 9-residue window centered on each residue. This feature vector is fed into a SVM trained on a large dataset which classifies the residue-residue pair.

Both of our predictors participated in the most recent rounds of CASP (CASP8 and CASP9) and ranked among the top residue-residue contact predictors [97]. As an additional assessment, we evaluated both NNcon and SVMcon on all CASP9 targets. Table 2 shows the accuracy for medium and long range predicted contacts. Here, two amino acid residues are said to be in contact if the distance between their C_β_ atoms (C_α_ for glycine) in the experimental structure is less than 8 Å. Long range contacts are defined as residues in contact whose separation in the sequence is greater than or equal to 24 residues. Medium range contacts are defined by interacting residues which are 12 to 23 residues apart in the sequence. These definitions were used in accordance with previous studies and CASP residue-residue contact assessments [97,98]. A common evaluation metric for residue-residue contact predictions is the accuracy of the top *L*/5 or *L*/10 predictions where *L* is the length of the protein in residues and the predictions are ranked using a score provided for each prediction. Accuracy is defined as the number of correctly predicted residue-residue contacts divided by the total number of contact predictions considered. For medium range contacts, NNcon and SVMcon are capable of achieving accuracies at or above 35% when considering the top L/10 predictions and accuracies near 31% when considering the top L/5 predictions. For long range contacts, SVMcon performed notably better on the CASP9 targets with accuracies of 27% and 24% for the top L/10 and L/5 predictions, respectively, while NNcon obtained accuracies of 21% and 18%.

**Table 2:** Accuracy for NNcon and SVMcon contact predictions on all CASP9 targets

**Predictor**	**medium range contacts (12 < = seq. separation < 24)**		**long range contacts (seq. separation > = 24)**		
	**top L/10**	**top L/5**	**top L/10**	**top L/5**	**top L**
SVMcon	.35	.32	.27	.24	.14
NNcon	.36	.31	.21	.18	.11

#### *DIpro2.0 for protein disulfide bond prediction*

DIpro2.0 is a tool that uses kernel methods, two-dimensional recursive neural networks, and weighted graph matching for large-scale protein disulfide bridge prediction [99,100]. Given a protein sequence, it can predict if a cysteine in the protein participates in a disulfide bond and how bonding cysteines are connected. The method can handle proteins with arbitrary number of disulfide bonds. Benchmarked on a large disulfide bond data set [99], the specificity and sensitivity of classifying individual residues as bonded or non-bonded are 87% and 89%, respectively, and the accuracy of overall disulfide connectivity pattern prediction is 51%. Some other disulfide bond prediction tools are DiANNA [47], GDAP [48], and CYSPRED [49].

#### *BETApro1.0 for protein beta-sheet structure prediction*

BETApro1.0 integrates two-dimensional recursive neural networks and graph algorithms with protein sequence profiles and predicted structural features (e.g. secondary structure and relative solvent accessibility) to predict specific beta residue pairs, beta strand pairs, strand alignments, strand pairing direction, and beta-sheet topology for beta sheets in a protein [101]. BETApro1.0 was evaluated on a large dataset using different standard measures [101]. At the break-even point, the specificity and sensitivity of beta-residue pairing predictions is 41%. At 59% specificity, the sensitivity of beta strand pairing predictions is 54%. Some other beta-sheet prediction tools are BETAWRAP [50], SVM-BetaPred [44], and BETTY [51].

### 3D structure prediction and evaluation tools

#### *MULTICOM for tertiary structure prediction*

MULTICOM [102], an automated multi-level combination method, combines complementary and alternative templates, alignments, and models to predict protein tertiary structures. Several implementations of this approach with minor differences were tested in the last two Critical Assessments of Techniques for Protein Structure Predictions (CASP8 and CASP9) in 2008 and 2010, respectively [102]. One significant improvement on multi-template combination benchmarked in CASP9 is to check the structural consistency between multiple template candidates. This procedure avoids potential atom clashes caused by conflicting structural conformations from inconsistent templates. The structural similarity of a pair of query-template alignments was checked by comparing the structures of two templates after they are aligned to the same regions of the query using TM-Align [103]. Only structurally similar query-template alignments are combined. Both MULTICOM-server and MULTICOM-human predictors were ranked among the best in CASP8 and CASP9.

**Figure 4:**
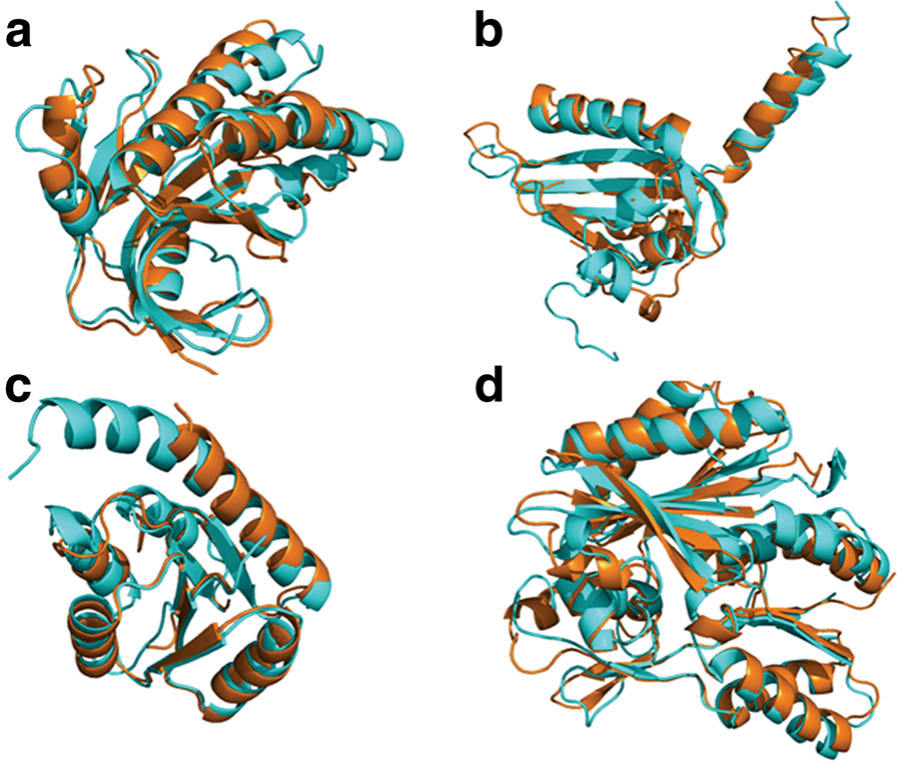
**Superimpositions of predicted models (blue) and native structures (orange) of four CASP9 targets.** (**A**) T0520, TM-Score = 85, (**B**) T0527, TM-Score = 74, (**C**) T0634, TM-Score = 88, (**D**) T0641, TM-Score = 91.

Table 3 illustrates the evaluation results of one MULTICOM server predictor and one MULTICOM human predictor. The evaluation was conducted on 107 CASP9 targets, whose native structures were downloaded from the Protein Data Bank [104]. We used TM-Score [103] to compare predicted models with native structures to calculate their similarity scores in terms of both GDT-TS score [105] and TM-Score [103]. GDT-TS scores or TM-Scores are in the range [0, 100], where 0 means completely different and 100 exactly the same. Generally, a TM-Score of 50 indicates a reasonable model with largely correctly predicted topology and a score greater than 80 is a high-quality model. On average, the GDT-TS score and TM-Score of the first MULTICOM server models are 59.28 and 66.76, respectively, indicating the average quality of server models is good. The average score of MULTICOM-server models is 2–4 points lower than MULTICOM-human’s, one of the best CASP9 human predictors that made predictions by exploring the entire CASP9 model pool. This suggests that the automatically generated MULTICOM-server predictions are approaching the best performance among CASP9 models. Figure 4 shows good-quality models predicted by MULTICOM-server on four CASP9 targets.

**Table 3:** The average GDT-TS and TM scores of top-one and best-of-five models of MULTICOM predictors on 107 CASP9 targets

**Predictor**	**First Model**		**Best of Five**	
	**GDT-TS**	**TM-Score**	**GDT-TS**	**TM-Score**
MULTICOM (human)	63.14	70.53	64.41	71.85
MULTICOM (server)	59.28	66.76	62.02	69.29

#### *APOLLO for protein model quality assessment*

APOLLO is a software package that can predict global and residue-specific qualities of individual or multiple protein models without knowing native structures [106]. For an individual model, APOLLO uses a machine learning method (support vector machine) to predict its absolute global [107] and residue-specific qualities [106]. The absolute global quality of a model is the overall structural similarity between the model and its native structure in terms of GDT-TS score, whereas the absolute residue-specific qualities are the structural deviations at each residue position in terms of Angstrom (Å). The features used in the machine learning algorithm include amino acid sequence and the differences between predicted (predicted from amino acid sequence) and parsed (parsed from protein model) secondary structures, solvent accessibilities, and residue-residue contact probabilities. For multiple models, APOLLO uses a pair-wise comparison method to predict their relative global qualities [108]. This algorithm performs a full pair-wise comparison of each model against all the others by the structural alignment program TM-Score [103]; and the average structural similarity scores are used as the predicted global qualities. APOLLO also employs a hybrid approach to refine absolute quality scores. It selects the top five models ranked by initial quality scores as reference models and then superimposes every model with each of the reference models by TM-Score [109]. The average GDT-TS score resulted from the superimpositions is used as the predicted global quality.

We evaluated the APOLLO software package on the models of 107 valid CASP9 targets whose experimental structures were available in the Protein Data Bank [104]. For global quality prediction, the average Pearson’s correlations between predicted and real quality scores of pair-wise, hybrid, and machine learning methods are 0.917, 0.870, and 0.671, respectively [106]. For residue-specific quality prediction, APOLLO has an average error deviation of 2.60 and 3.18 Å on the residues whose actual distances to the native are < = 10 and 20 Å, respectively [106].

### Other protein bioinformatics tools

#### *MUpro1.0 for protein mutation stability prediction*

MUpro1.0 [110] is a tool using support vector machines to predict protein stability changes for single amino acid mutations. It can predict the amount of the energy change caused by an amino acid mutation from a protein sequence, a protein structure, or both. MUpro1.0 was evaluated on a large dataset of single amino acid mutations [110]. It predicted the direction (positive versus negative) of the mutation-induced energy changes at 84% accuracy. The method can also reliably predict the absolute value of an energy change. Some mutation stability prediction tools are PoPMuSiC [111], SDM [112], I-Mutant2.0 [113], and CUPSAT [114].

#### *SeqRate for protein folding rate prediction*

SeqRate [115] is a sequence-based tool for large-scale protein folding rate prediction. It uses a Support Vector Machine regression method with a set of features derived from protein sequences alone to make predictions. The tool can predict both folding kinetic types and real-value folding rates. The folding kinetic type prediction accuracy of SeqRate on a standard benchmark is 80% [115].

#### *MSACompro1.2.0 for protein multiple sequence alignment with predicted structural features*

MSACompro1.2.0 [116] is a new tool that integrates predicted secondary structure, solvent accessibility, and contact map information with protein sequences to improve protein multiple sequence alignment. MSACompro1.2.0 was evaluated on the BAliBASE 3.0 datasets [117], yielding an average alignment Sum of Pair score (SP score) of 88.85 and the average alignment True Column score (TC score) of 61.31. The results showed that incorporating protein structural features into multiple sequence alignment improves alignment accuracy over existing tools without using structural features.

#### *HMMEditor for visualization of hidden Markov models of protein sequence family*

HMMEditor [118] is a visual, interactive editor for visualizing and manipulating profile Hidden Markov Models of a protein family. It provides a series of functions to visualize the profile HMM architecture, transition probabilities, and emission probabilities. It also allows users to align a sequence against the profile HMM and visualize the corresponding Viterbi path.

### Software packages, web services, documentation, and user support

**Figure 5:**
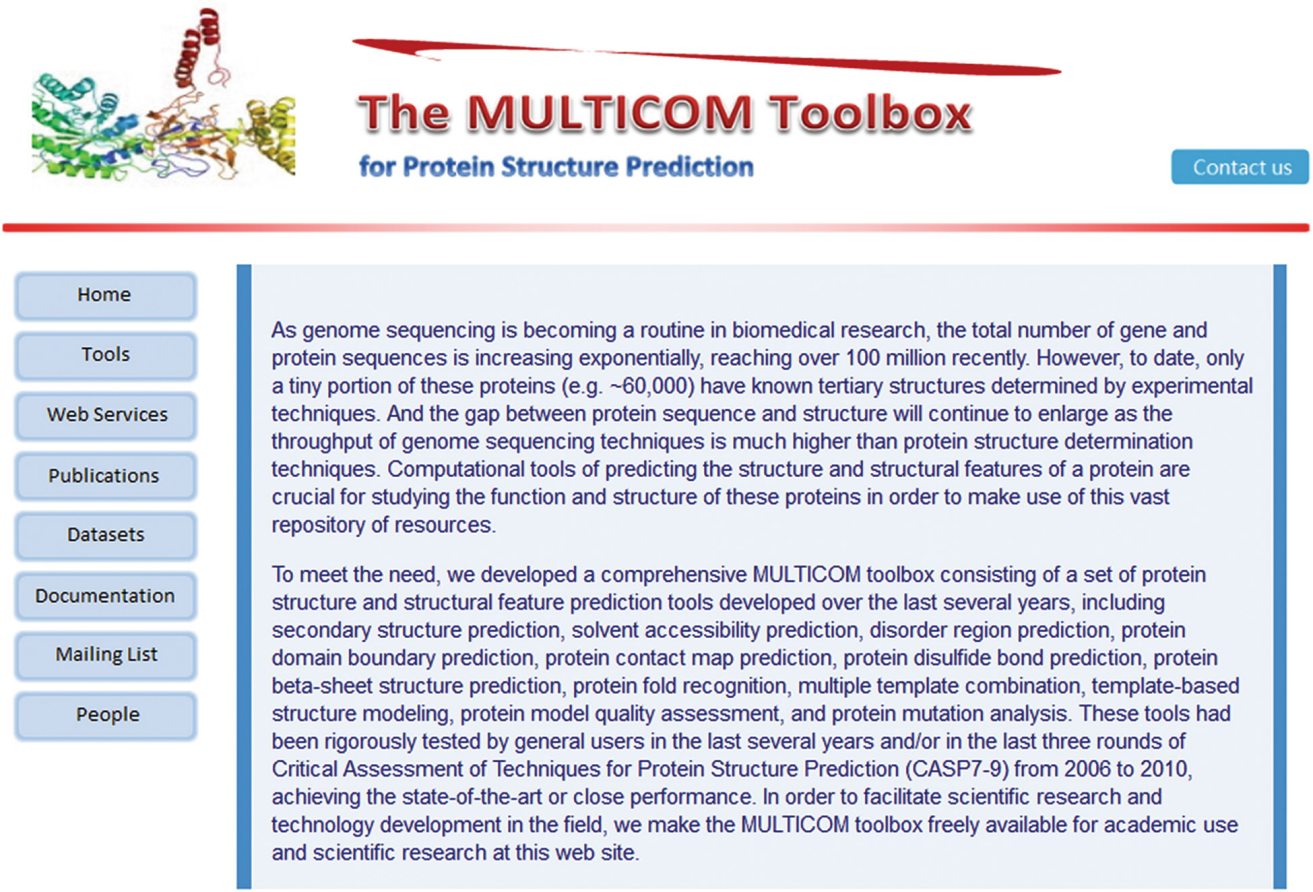
The MULTICOM toolbox web site.

Most tools in the MULTICOM toolbox are available as both downloadable software packages and online web services at the *one-stop* web site http://sysbio.rnet.missouri.edu/multicom_toolbox/ (Figure 5). Some tools that are only available as web services will be released as software packages in the near future. The documentation and relevant publications of these tools are also available at the same web site. Table 4 summarizes the availability and running environment of the MULTICOM tools.

**Table 4:** The availability and running environment of the MULTICOM tools

**Tools**	**Software Package**	**Source Code**	**Web Service**	**Platform**	**Documentation**
PSpro2.0	Yes	Yes	Yes	Linux, Browser	PDF, HTML
PreDisorder1.1	Yes	Yes	Yes	Linux, Browser	PDF, HTML
DoBo			Yes	Browser	PDF, HTML
NNCon	Yes		Yes	Linux, Browser	PDF, HTML
SVMcon	Yes		Yes	Linux, Browser	PDF, HTML
DIpro2.0	Yes	Yes		Linux	PDF, HTML
BETApro1.0	Yes	Yes	Yes	Linux, Browser	PDF, HTML
MULTICOM			Yes	Browser	PDF, HTML
APOLLO	Yes	Yes	Yes	Linux, Browser	PDF, HTML
MUpro1.0	Yes	Yes	Yes	Linux, Browser	PDF, HTML
SeqRate	Yes		Yes	Linux, Browser	PDF, HTML
MSACompro1.2.0	Yes			Linux	PDF, HTML
HMMEditor	Yes		Yes	Linux, Browser, Unix, Windows	PDF, HTML

The MULTICOM toolbox has been implemented in different programming languages including C++, Java, and Perl. The tools have been extensively tested on the Linux platform. We expect to gradually release some standalone tools for other popular platforms such as Windows and Mac. Most of the tools in the toolbox are available as online web services, which makes it easy for users to make predictions on a small scale without a need to install the software. The web interface is generally simple and intuitive and requires a minimum amount of information from the user. The results may be sent to users by email or be presented in the browser. Most tools are also available as software packages that can be downloaded by users for large-scale prediction or other purposes. In general, installing these tools is straightforward and often only requires unzipping the software, setting a few paths in a configuration file, and running a configuration script. The package of each tool includes a readme file that contains both installation instructions and a quick guide on using the tool. One or more test examples with expected results are often provided with the package for users to test an installation.

In order to facilitate the use of the tools, the user manuals for these tools have been developed in PDF and HTML format and are available at the MULTICOM web site. The user manuals usually include step-by-step installation instructions, application examples, references to more technical documents, and frequently asked questions (FAQ) and solutions. In order to better serve users and gather community feedback to improve the toolbox, a mailing list is created. After subscribing the MULTICOM mailing list (multicom_toolbox@googlegroups.com), a user can post a message to the mailing list and view the collection of all prior postings. The technical support of the MULTICOM toolbox regularly reads the message postings and answers questions. Collected improvements will be released in future versions of the toolbox.

## Conclusion

We developed a comprehensive MULTICOM toolbox consisting of a number of protein structure and structural feature prediction tools. These tools have been extensively tested and used internally and externally during the last several years yielding good performance. All the tools are freely available as software packages and/or online web services for academic use and scientific research at the MULTICOM web site. This makes them useful for large-scale annotation of structure and function of vast protein sequence resources generated in the genomic era. In the future, we will continue to improve the performance, usability, and documentation of these tools, make them available to more platforms (e.g. Windows and Mac), and add new protein structure and function prediction tools into the toolbox. Improvements and new developments will be released on the MULTICOM toolbox web site.

## Competing interests

The authors declare that they have no competing interests.

## Authors’ Contributions

JC conceived the system. JC, JL, ZW, JE, XD designed, developed and tested the system. JC, JL, ZW, JE, XD authored, edited and approved the manuscript. All authors read and approved the final manuscript.
